# The Sphingolipid Asset Is Altered in the Nigrostriatal System of Mice Models of Parkinson’s Disease

**DOI:** 10.3390/biom12010093

**Published:** 2022-01-06

**Authors:** Victor Blokhin, Maria Shupik, Ulyana Gutner, Ekaterina Pavlova, Albert T. Lebedev, Olga Maloshitskaya, Vsevolod Bogdanov, Sergey Sokolov, Alice Alessenko, Michael Ugrumov

**Affiliations:** 1 Laboratory of Neural and Neuroendocrine Regulations, Koltzov Institute of Developmental Biology of the Russian Academy of Sciences, 119334 Moscow, Russia; victor.blokhin@hotmail.com (V.B.); guchia@gmail.com (E.P.); vse-bogd@yandex.ru (V.B.); 2 Institute of Biochemical Physics Named after N.M. Emanuel of the Russian Academy of Sciences, 119334 Moscow, Russia; mariashupik@gmail.com (M.S.); uliana.goutner@gmail.com (U.G.); maloshitskaya@mail.ru (O.M.); 3 Department of Organic Chemistry, Lomonosov Moscow State University, 119991 Moscow, Russia; mocehops@yandex.ru (A.T.L.); sokolovsa48@gmail.com (S.S.)

**Keywords:** mice, 1-methyl-4-phenyl-1,2,3,6-tetrahydropyridine, modeling of Parkinson’s disease, dopaminergic nigrostriatal system, sphingolipids, enzymes, gene expression, mass spectrometry, high-performance liquid chromatography, real-time PCR

## Abstract

Parkinson’s disease (PD) is a neurodegenerative disease incurable due to late diagnosis and treatment. Therefore, one of the priorities of neurology is to study the mechanisms of PD pathogenesis at the preclinical and early clinical stages. Given the important role of sphingolipids in the pathogenesis of neurodegenerative diseases, we aimed to analyze the gene expression of key sphingolipid metabolism enzymes (ASAH1, ASAH2, CERS1, CERS3, CERS5, GBA1, SMPD1, SMPD2, UGCG) and the content of 32 sphingolipids (subspecies of ceramides, sphingomyelins, monohexosylceramides and sphinganine, sphingosine, and sphingosine-1-phosphate) in the nigrostriatal system in 1-methyl-4-phenyl-1,2,3,6-tetrahydropyridine (MPTP) mouse models of the preclinical and clinical stages of PD. It has been shown that in PD models, the expression of five of the nine studied genes (CERS1, CERS5, ASAH1, ASAH2, and GBA1) increases but only in the substantia nigra (SN) containing dopaminergic cell bodies. Changes in the expression of enzyme genes were accompanied by an increase in the content of 7 of the 32 studied sphingolipids. Such findings suggest these genes as attractive candidates for diagnostic purposes for preclinical and clinical stages of PD.

## 1. Introduction

After Alzheimer’s disease, Parkinson’s disease (PD) is the second-most common and severe socially significant neurodegenerative disease, the effectiveness of treatment for which is limited. It is generally accepted that this is due to late diagnosis and onset of treatment for this disease [1,2,3]. Indeed, the first motor symptoms, which are used to diagnose PD, appear many years (up to 30 years) after disease onset, following the death of most of the dopaminergic neurons of the nigrostriatal system of the brain, a key link in the regulation of motor function [4,5]. Based on this concept, the main hope for increasing the effectiveness of PD treatment is associated with the development of early (preclinical) diagnosis and preventive neuroprotective therapy [3,6]. Solving these issues will probably make it possible to slow down the death of neurons, primarily dopaminergic ones, and thus significantly prolong the period of comfortable life, as well as of the social and physical activity of the patient.

In accordance with the methodology of translational medicine, the development of technologies for early diagnosis and preventive treatment of PD should be based on fundamental knowledge of the molecular mechanisms of the pathogenesis of this disease, primarily the mechanisms of neurodegeneration and neuroplasticity. In recent years, experimental and clinical data have been accumulating, indicating that along with the known triggers of neurodegeneration in PD, such as neuroinflammation and the neurotoxic effect of pathologically altered, aggregated alpha-synuclein [7,8], an important role in the pathogenesis of PD is played by disturbed sphingolipid metabolism [9,10]. Specifically, in PD, a mutation in the gene of glucocerebrosidase, an enzyme that cleaves the sphingolipid glucosylceramide to glucose and ceramide, was discovered [11]. In addition, in the literature, the first evidence was obtained that sphingolipids such as ceramides, sphingomyelins, monohexosylceramides, sphinganine, sphingosine, and sphingosine-1-phosphate, are involved in apoptosis, autophagy, and neuroinflammation [10,12,13,14,15]. It is believed that impaired ceramide metabolism is directly related to the development of alpha-synucleinopathy [16] and, as a consequence, to the formation of Lewy bodies, a key PD marker [17,18]. However, under certain conditions, sphingolipids can also participate in reparative processes [10,19].

Existing pioneering studies of the role of sphingolipids in the molecular mechanisms of PD pathogenesis open up broad prospects for the development of new methods for and the improvement of existing approaches to the diagnosis and treatment of PD. So, recently, the first attempts have been made to translate fundamental knowledge about systemic impairment of sphingolipid metabolism in PD into the development of differential diagnosis and treatment for this disease, based on a search for biomarkers in the body fluids (cerebrospinal fluid and blood) [20,21,22]. In addition, it was found that changes in sphingolipid metabolism increase the risk of PD, and correcting sphingolipid levels by regulating the activity of enzymes involved in sphingolipid metabolism can either slow down or prevent the development of this pathology [23].

Considering that studies of PD pathogenesis are extremely limited in patients and are impossible at the preclinical stage, studies on experimental models are of particular importance. Therefore, the aim of this study was to analyze the gene expression of key enzymes of sphingolipid metabolism and to assess changes in sphingolipid metabolism in mice using original neurotoxic models of the preclinical and early clinical stages of PD. To achieve this goal, the following objectives were set: (1) to use mouse models of PD at the preclinical and clinical stages, obtained by systemic administration of 1-methyl-4-phenyl-1,2,3,6-tetrahydropyridine (MPTP), which is converted in the body into MPP +, a dopaminergic neuronal toxin; (2) to assess changes in the content of sphingolipids in the nigrostriatal system of the brain (striatum and substantia nigra (SN)), such as ceramides, sphingomyelins, monohexosylceramides, sphinganin, sphingosine, and sphingosine-1-phosphate, which play a key role in neurodegeneration and neuroplasticity; (3) to evaluate the gene expression of some key enzymes of sphingolipid metabolism, such as ceramide glucosyltransferase; ceramide synthase 1, 3, 5; acid ceramidase 1; neutral ceramidease 2; glucocerebrosidase 1; sphingomyelin phosphodiesterase 1; and sphingomyelin phosphodiesterase 2; and (4) to carry out a comparative analysis of the expression of genes for enzymes of sphingolipid metabolism in different areas of the nigrostriatal system—in the striatum and in the SN, in mice using neurotoxic models of preclinical and clinical stages of PD.

## 2. Materials and Methods

### 2.1. Animals and Experiments

In this study, 102 male C57Bl/6J mice, aged from 2 to 2.5 months, weighing 23–25 g, were used. The animals were kept in a vivarium with a 12 h day and night cycle and free access to food and water. All manipulations with the animals were carried out in accordance with national and international requirements and the rules approved by the Animal Care Committee of the Koltzov Institute of Developmental Biology of the Russian Academy of Sciences (protocol no. 40, dated 17 September 2020).

To simulate the preclinical stage of PD, mice (n = 34) were injected subcutaneously once with MPTP (Sigma-Aldrich, Darmstadt, Germany) in 0.9% NaCl (saline) at a dose of 18 mg/kg. To simulate the clinical stage of PD, mice (n = 34) were injected subcutaneously three times with MPTP in saline at a single dose of 10 mg/kg with an interval of 2 h between injections. Control mice (n = 34) were injected subcutaneously with saline.

**Figure 1:**
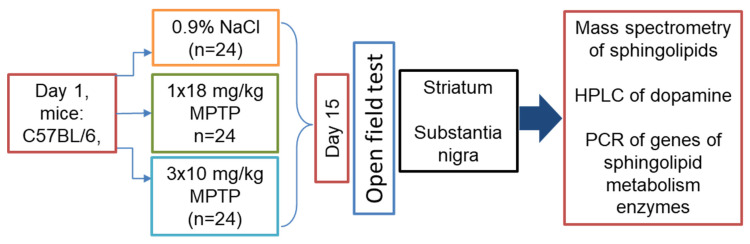
Design of experiments for modeling Parkinson’s disease at the preclinical and clinical stages in C57BL/6 mice by subcutaneous administration of 1-methyl-4-phenyl-1,2,3,6-tetrahydropyridine (MPTP), once at a single dose of 18 mg/kg and three times at a single dose of 10 mg/kg, with a 2-h interval between injections, respectively. In the controls, saline was administered.

Two weeks after the administration of MPTP or saline, in mice that received MPTP once at a dose of 18 mg/kg (n = 8), in mice that received MPTP three times in a single dose of 10 mg/kg (n = 8), and in control mice that received 0.9% NaCl (n = 8), motor behavior was assessed in the open-field test for 6 min (Figure 1). A PhenoMaster animal behavior analysis device (TSE Systems, Bad Homburg, Germany) was used for this.

All mice were anesthetized with isoflurane (Kent Scientific, Torrington, CT, USA) 2 weeks after the administration of MPTP or saline. The animals were decapitated under anesthesia, and the brains were removed. Then we excised the rostrodorsal striatum and the SN under the binocular loupe in accordance with the atlas of the mouse brain [24]. Pieces of tissue were weighed, frozen in liquid nitrogen, and stored at −70 °C until high-performance liquid chromatography with electrochemical detection (HPLC-ED), real-time PCR, and mass spectrometry were performed.

### 2.2. Methods

#### 2.2.1. High-Performance Liquid Chromatography with Electrochemical Detection

The samples for measuring dopamine were prepared by solid-phase extraction using aluminum oxide. The dopamine content in the samples was determined by HPLC-ED according to the previously described protocol [25]. To measure dopamine, the frozen tissue was thawed and homogenized with an ultrasonic homogenizer at 4 °C in 400 μL (for the striatum) or 100 μL (for the SN) of 0.1 M HClO_4_ and 250 pM 2,3-dihydroxybenzoic acid. Then, part of the homogenate was taken to measure the protein concentration using the BCA Protein Assay Kit in accordance with the manufacturer’s instructions (Thermo Scientific Pierce, Waltham, MT, USA). The remainder of the homogenate was centrifuged for 20 min at a speed of 18,000× *g* at +4 °C. The supernatant was taken for further analysis.

Dopamine was determined on a reversed-phase column (100 × 4 mm ReproSil-Pur C18, 3 μm; Dr. Maisch, Ammerbuch, Germany). The mobile phase was 0.1 M citrate-phosphate buffer containing 0.25 mM sodium octanesulfonate, 0.1 M EDTA, and 6% acetonitrile (pH 2.55) and running at a speed of 1 mL/min. The potential of substances leaving the column was determined using an electrochemical detector DECADE II (Antec Leyden, Zoeterwoude, The Netherlands) at 2 nA. The peaks of catecholamines and their metabolites were identified by the time of release relative to the standard solution. The retention time for 3-dihydroxybenzoic acid used as an internal standard was 3 min and 15 s, whereas for dopamine, it was 4 min and 31 s. The content of catecholamines was calculated as the ratio of the peak areas in the sample to the standards. Peak areas were measured using LabSolutions software (Shimadzu, Kyoto, Japan). Dopamine content was adjusted to tissue weight.

#### 2.2.2. Real-Time PCR

RNA was extracted from tissue using TRI-Reagent (Sigma-Aldrich, Darmstadt, Germany) according to the manufacturer’s instructions. For precipitation of RNA, 1 μg of glycogen (Thermo Fisher Scientific, Waltham, MT, USA) was used per sample of the SN. RNA concentration was determined using Nanodrop 8000. For cDNA synthesis, only samples with RNA absorption coefficients A260/280 ranging from 1.9 to 2.1 were used. Total RNA was treated with RNase-free DNase I (Thermo Fisher Scientific, Waltham, MT, USA) to remove residual genomic DNA. For reverse transcription for the synthesis of cDNA, we took 1 µg of RNA from striatum samples and 0.5 µg of RNA from SN samples. For conducting reverse transcription, a RevertAid H Minus First Strand cDNA Synthesis Kit (Thermo Fisher Scientific, USA) with random hexamer primers was used in accordance with the manufacturer’s instructions (Thermo Fisher Scientific, Waltham, MT, USA). The reaction lasted 60 min at 42 °C and was stopped by heating at 70 °C for 10 min, followed by cooling the samples on ice. For quantitative PCR analysis, we used 0.5 μg of cDNA per reaction and a qPCRmix-HS SYBR + LowRox kit (catalog no. PK156L, Evrogen, Moscow, Russia) in accordance with the manufacturer’s instructions. The primers used (Evrogen, Russia) are presented in Table 1. All the reactions (40 cycles) were performed in a QuantStudio 12 K Flex real-time PCR system (Applied Biosystems, Carlsbad, CA, USA). Gene expression levels are expressed as ΔΔCt values normalized to the GAPDH level. The following formula was used for calculating ΔΔCt:

∆∆Ct = ∆Ct(Sample) − ∆Ct(Control average)

#### 2.2.3. Mass Spectrometry of Sphingolipids

Lipids were extracted from the striatum and the SN according to the Bligh and Dyer method [26]. Lipid analysis was performed by HPLC/MS on a TSQ Endura device (Thermo Fisher Scientific, Waltham, MT, USA) equipped with an EclipsePlusC8 HPLC column (Agilent, Palo Alto, CA, USA) in multiple reaction monitoring (MRM) mode at a collision cell pressure of 2.0 mTorr, with a resolution of 1.2 Da at Q1 and Q3. The ionization method was ESI; the capillary voltage was 3.5 kV. For ceramides, the initial protonated and dehydrated molecules were fragmented at an energy of 20 V to an ion with *m*/*z* 264.2 at a dwell time of 30 ms. For SM, the fragmentation of the initial protonated molecules was carried out at an energy of 20 V to an ion with *m*/*z* 184.1 at a dwell time of 30 ms. For sphingosine and sphinganine, the fragmentation of protonated molecules was performed at energies of 12.5 eV to form ions with *m*/*z* 252.3 and 266.2, respectively, at a dwell time of 30 ms. For sphingosine-1-phosphate, the protonated molecules were fragmented at 20 eV to form ions with *m*/*z* 264.2. For monohexosylceramides, the initial protonated and dehydrated molecules were fragmented at an energy of 35 V to an ion with *m*/*z* 264.2 at a dwell time of 30 ms.

When determining sphingosine and sphinganine, the following ionization source parameters were used: heater temperature 300 °C, capillary temperature 340 °C, sheath gas flow 45 arb, aux gas flow 13 arb, and sweep gas flow 1 arb.

The following ionization source parameters were used in the detection of ceramides, sphingomyelins, sphingosine-1-phosphate, and monohexosylceramides: heater temperature 300 °C, capillary temperature 350 °C, sheath gas flow 50 arb, aux gas flow 15 arb, and sweep gas flow 2 arb.

Calibration curves were obtained in the following concentration ranges: SM16:0 (RT 14,5 min) 50–10,000 ng/mL; SM18:0 (RT 15,3 min) 50–10,000 ng/mL; CER d18:1/16:0 (RT 14,4 min) 5–200 ng/mL; CER d18:1/18:0 (RT 14,5 min) 5–200 ng/mL; CER d18:1/18:1 (RT 14,7 min) 4–160 ng/mL, d18:1/24:0 (RT 16,9 min) 10–2000 ng/mL, d18:1/24:1 (RT 16,4 min) 5–1000 ng/mL; sphinganine (RT 6,4 min) 2–60 ng/mL, sphingosine (RT 6,2 min) 2–60 ng/mL; glucosylceramide d18:1/18:0 (RT 14,5 min) 2–40 ng/mL; and sphingosine-1-phosphate C17 (RT 7,4 min) 10–2000 ng/mL. All these standard substances as well as galactosylceramide d18:1/18:0, sphingosine-d7, and sphingosine-1-phosphate were obtained from Avanti (Avanti Polar Lipids, Birmingham, AL, USA). For each lipid, the ratio of the area under the chromatographic peak of this molecule to the area of the chromatographic peak from the standard molecule was calculated (using the external standard method).

Chromatographic separation of lipids was performed on an Ultimate 3000 HPLC system (Thermo Fisher, Bremen, Germany) with an Eclipse Plus C8 3.0 × 150 mm × 3.5 ϻm column (Agilent; USA) at 50 °C and a flow rate of 500 mL/min (for sphinganine and sphingosine) and at 35 °C and a flow rate of 400 mL/min (for ceramides, sphingomyelins, sphingosine-1-phosphate, and monohexosylceramides). When determining sphinganine and sphingosine, the following mobile phases were used: phase A, water + 0.1% (*v*/*v*) HCOOH; phase B, methanol + 0.1% (*v*/*v*) HCOOH (0.7 min 55% of phase B, 100% of phase B by 6.7 min and up to 18 min, 55% of phase B by 20 min and up to 25 min). In the determination of ceramides, sphingomyelins, sphingosine-1-phosphate, and monohexosylceramides, the following mobile phases were used: phase A, water + 0.1% (*v*/*v*) HCOOH + 1 m M HCOONH4; phase B, methanol + 0.1% (*v*/*v*) HCOOH + 1 mM HCOONH4 (0 min 75% of phase B, 100% of phase B by 14 min and up to 24 min, 75% of phase B by 26 min and up to 30 min).

#### 2.2.4. Statistical Analysis

Statistical data analysis was performed using GraphPad Prism software version 6.0 (GraphPad Software, San Diego, CA, USA) and Microsoft Excel version 16.37 (Microsoft, Albuquerque, NM, USA). Quantitative changes are presented as the mean ± SEM. The Shapiro–Wilk test was performed to confirm the normal distribution of all quantitative variables. To prove the hypothesis of the equality of variances, Levene’s test was performed. In the case of a normal distribution of data that did not meet the requirement of the equality of variances, the two groups were subjected to a two-sample *t*-test with unequal variances. If the data showed a normal distribution and had equal variances, we performed Student’s *t*-test (for two groups) or ANOVA (for three groups). For multiple group comparisons, Tukey’s test was performed. In other cases, the Mann–Whitney U test was performed for two groups and the Kruskal–Wallis H test for three groups. We considered *p* ≤ 0.05 as the criterion of significance.

## 3. Results

### 3.1. Motor Behavior

**Figure 2:**
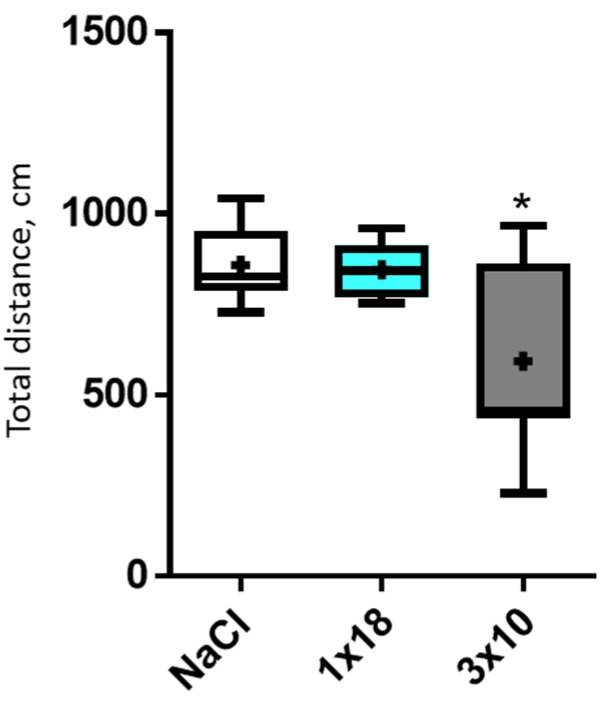
Motor behavior: distance traveled in the open-field test in mice 2 weeks after administration of MPTP once at a dose of 18 mg/kg (1 × 18), three times at a single dose of 10 mg/kg (3 × 10), and in the controls (NaCl). * *p* = 0.05, significant difference compared to the controls; “+”: average; “−”: median.

The distance traveled in the open-field test did not change in mice treated with MPTP at a single dose of 18 mg/kg compared with the controls (Figure 2). However, this parameter decreased in mice treated with MPTP three times at a single dose of 10 mg/kg (593 cm vs. 858 cm, *p* = 0.038, n = 8).

### 3.2. Dopamine Content in the Striatum and Substantia Nigra in Mice after MPTP Administration

**Figure 3:**
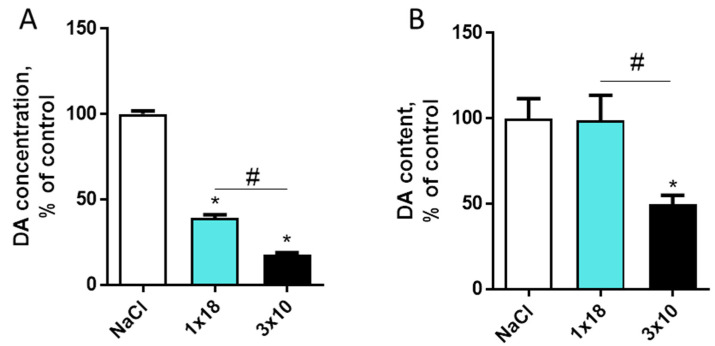
Changes in the dopamine (DA) concentration in the striatum (**A**) and changes in the dopamine content in the substantia nigra (**B**) in mice 2 weeks after subcutaneous administration of 1-methyl-4-phenyl-1,2,3,6-tetrahydropyridine (MPTP): once at a single dose of 18 mg/kg (1 × 18) or three times at a single dose of 10 mg/kg with a 2-h interval between injections (3 × 10), compared to the controls (saline), taken as 100%. * *p* < 0.05, significant difference compared to the controls, taken as 100%; # *p* < 0.05, differences between the groups 1 × 18 and 3 × 10.

After a single subcutaneous injection of MPTP at a dose of 18 mg/kg, the concentration of dopamine in the striatum decreased by 60% compared to the controls (saline) (Figure 3). In the group receiving MPTP three times at a single dose of 10 mg/kg at 2 h intervals, the decrease in dopamine in the striatum was 82% (Figure 3). In the SN, dopamine levels did not change following a single injection of MPTP, while they decreased by about 50% following a triple injection (Figure 3).

### 3.3. Concentration of Sphingolipids in the Nigrostriatal System in the Brain in Mice after MPTP Administration

#### 3.3.1. Concentrations of Sphingomyelins in the Striatum and Substantia Nigra in Mice Two Weeks after MPTP Administration

In mice that received MPTP at a single dose of 18 mg/kg or three times at a single dose of 10 mg/kg with a 2 h interval between injections, changes in the concentration of 12 molecular types of sphingomyelins were assessed in the striatum and in the SN: 14-0, 16-1, 16-0, 18-1, 18-0, 20-1, 20-0, 22-1, 22-0, 24-1, 24-0, and 26-1 versus the controls (saline). The concentration of individual molecular types of sphingomyelins in the control mice varied significantly both in the striatum and in the SN. In the striatum, the concentration ranged from 14 ng/10 mg tissue (22-0) to 2625 ng/10 mg tissue (20-0), and in the SN, it ranged from 14 ng/10 mg tissue (22-0) to 5915 ng/10 mg tissue (24-1) (Table S1).

**Figure 4:**
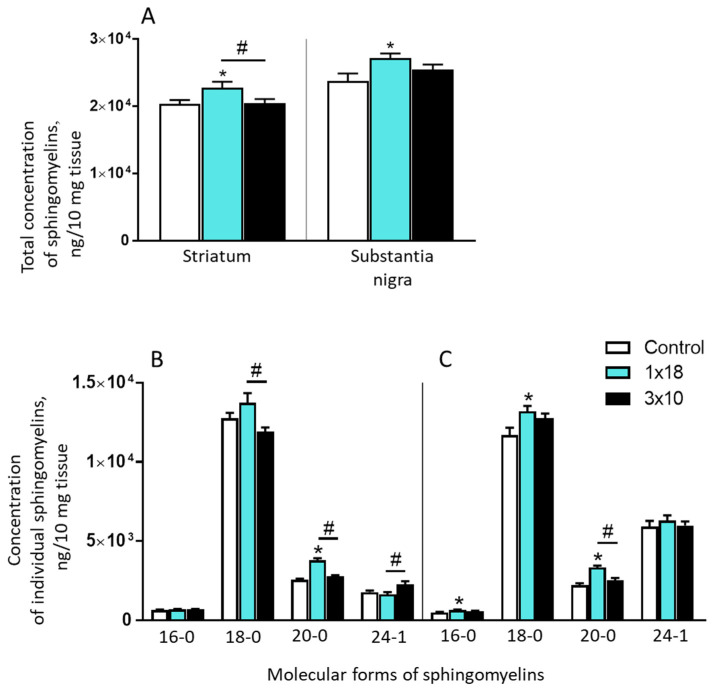
Total (cumulative) concentration of all measured sphingomyelins (14-0, 16-1, 16-0, 18-1, 18-0, 20-1, 20-0, 22-1, 22-0, 24-1, 24-0, and 26-1) in the striatum and substantia nigra (**A**) in mice 2 weeks after the administration of 1-methyl-4-phenyl-1,2,3,6-tetrahydropyridine (MPTP): once at a single dose of 18 mg/kg (1 × 18) and three times at a single dose of 10 mg/kg (3 × 10), as well as the concentration of individual sphingomyelins in the striatum (**B**) and in the substantia nigra (**C**) in the same experiments and in the controls (saline). * *p* < 0.05, significant differences compared to the controls; # *p* < 0.05, significant differences between the groups 1 × 18 and 3 × 10.

With a single dose of MPTP, the total (cumulative) concentration of all measured sphingomyelins increased in the striatum and in the SN compared to the controls (Table S1; Figure 4A). Evaluating the change in the concentration of individual sphingomyelins, it was shown that with a single injection of MPTP at a dose of 18 mg/kg, in the striatum, the concentration of only one of them increased: 20-0 (Figure 4B). However, in the SN in the same animals, the concentration of three sphingomyelins increased: 16-0, 18-0, and 20-0 (Figure 4C).

When MPTP was injected three times, the total concentration of sphingomyelins in mice in the striatum and in the SN did not change compared to the controls (Figure 4A). In this experiment, there were also no changes in the concentration of individual molecular forms of sphingomyelins in the striatum (Figure 4B) and in the SN (Figure 4C; Table S1).

#### 3.3.2. Concentration of Ceramides in the Striatum and Substantia Nigra in Mice after MPTP Administration

In mice that received MPTP at a single dose of 18 mg/kg or three times at a single dose of 10 mg/kg, the concentration of 11 molecular types of ceramides was assessed: 14-0, 16-1, 16-0, 18-1, 18-0, 20-0, 22-1, 22-0, 24-1, 24-0, and 26-1 versus the controls. The concentration of different types of ceramides varied significantly in the controls, both in the striatum and in the SN. In the striatum, it ranged from 1.42 ng/10 mg tissue (26-1) to 1078.46 ng/10 mg tissue (18-0), and in the SN, it ranged from 1.67 ng/10 mg tissue (26-1) up to 479.57 ng/10 mg tissue (18-0). However, in the controls, the total (cumulative) concentration of all measured ceramides in the striatum was approximately twice that in the SN (Table S2).

**Figure 5:**
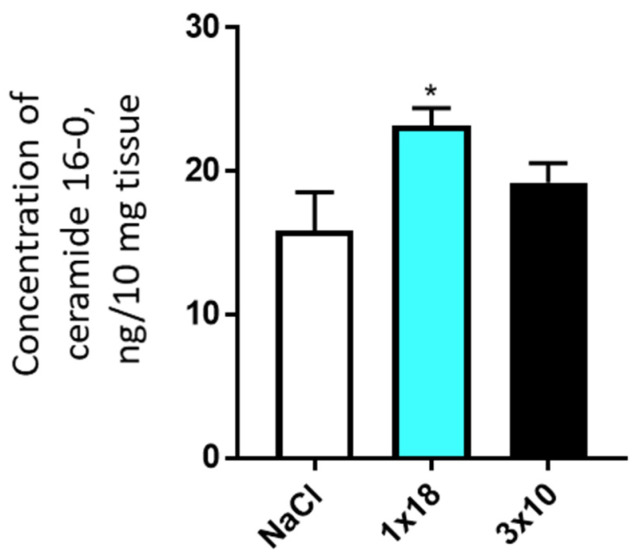
Concentration of ceramide 16-0 in the substantia nigra of mice 2 weeks after the administration of 1-methyl-4-phenyl-1,2,3,6-tetrahydropyridine, once at a single dose of 18 mg/kg or three times at a single dose of 10 mg/kg. * *p* < 0.05, significant differences compared to the controls.

In both experiments, with single and triple administration of MPTP, there were no changes in the concentration of most of the studied ceramides in the striatum and in the SN as compared to the controls. The only exception was ceramide C16-0, the concentration of which was slightly increased in the SN in mice after triple administration of MPTP (Figure 5; Table S2).

#### 3.3.3. Concentrations of Sphingosine, Sphinganine, and Sphingosine-1-Phosphate in the Striatum and Substantia Nigra in Mice after MPTP Administration

**Figure 6:**
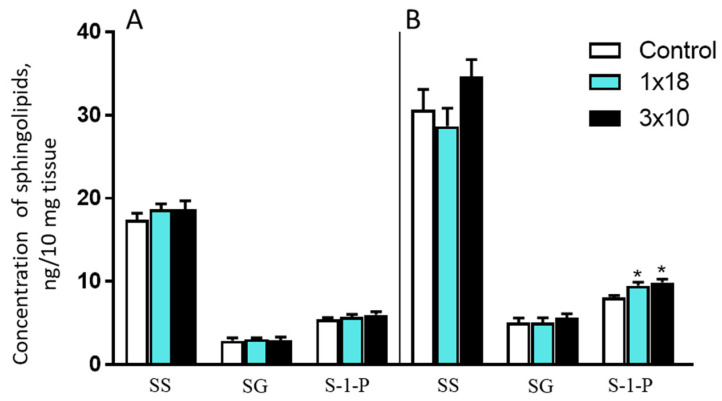
Concentration of sphingosine (SS), sphinganine (SG), and sphingosine-1-phosphate (S-1-P) in the striatum (**A**) and in the substantia nigra (**B**) in mice 2 weeks after the administration of 1-methyl-4-phenyl-1,2,3,6-tetrahydropyridine, once at a single dose of 18 mg/kg or three times at a single dose of 10 mg/kg. * *p* < 0.05, significant differences compared to the controls.

The concentrations of sphingosine, sphinganine, and sphingosine-1-phosphate varied significantly in the control mice, both in the striatum and in the SN (Figure 6). In both areas of the nigrostriatal system, the maximum concentration was displayed by sphingosine and the minimum one was shown by sphinganine. In experiments with single and triple administration of MPTP, only the concentration of sphingosine-1-phosphate changed and only in the SN. In both experiments, it increased in comparison with the controls by about 15% (Figure 6).

#### 3.3.4. Concentrations of Hexosylceramides in the Striatum and in the Substantia Nigra in Mice after MPTP Administration

In the controls, the concentration of the individual measured hexosylceramides—18-0, 20-0, 22-0, 24-0, and 24-1—varied significantly in both the striatum and the SN (Figure 5). Both in the striatum and in the SN, the maximum concentration was displayed by hexosylceramide 24-1 and the minimum one was exhibited by 20-0. In addition, in the controls, the concentration of each of the hexosylceramides in the striatum was 2–3 times lower than in the SN (Table S3).

**Figure 7:**
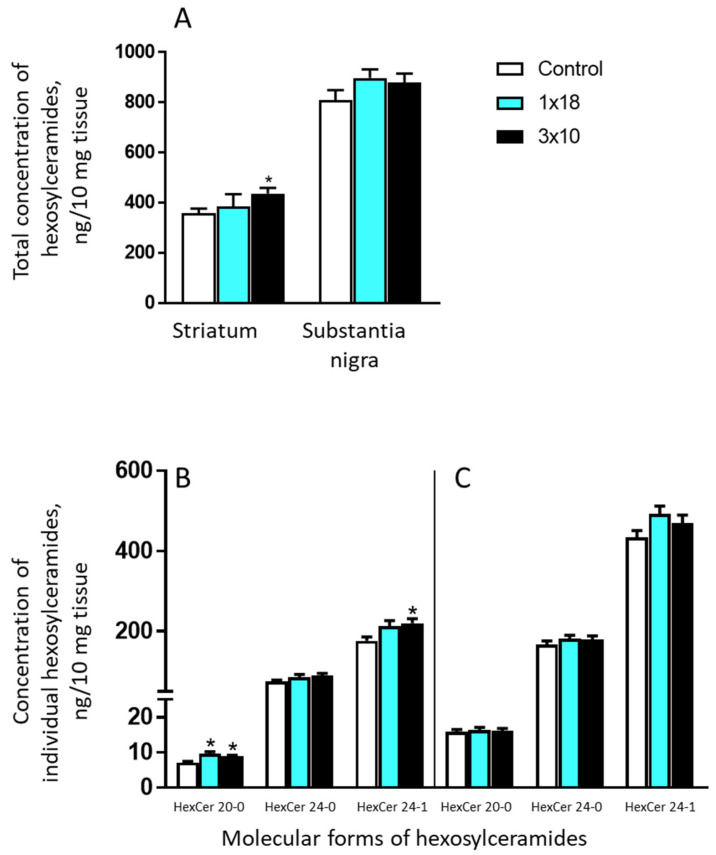
Total (cumulative) concentration of all measured hexosylceramides, 18-0, 20-0, 22-0, 24-0, and 24-1, in the striatum and in the substantia nigra (**A**) in mice 2 weeks after the administration of 1-methyl-4-phenyl-1,2,3,6-tetrahydropyridine once at a single dose of 18 mg/kg (1 × 18) or three times at a single dose of 10 mg/kg (3 × 10), as well as the concentration of individual hexosylceramides, 20-0, 24-0, and 24-1, in the striatum (**B**) and in the substantia nigra (**C**) in the same experiments and in the controls (saline). * *p* < 0.05, significant differences compared to the controls.

In both models, a change in the concentration of hexosylceramides was observed only in the striatum. Thus, with a single injection of MPTP, there was a slight increase in hexosylceramide 20-0 both in the striatum and in the SN (Figure 5). In this case, the total concentration changed insignificantly. After triple administration of MPTP, an increased level of hexosylceramide 20-0 was maintained. In addition, the concentration of hexosylceramide 24-1 increased, which generally led to an increase in the total concentration of hexosylceramides both in the striatum and in the SN (Figure 7).

### 3.4. Expression of Genes of Sphingolipid Metabolism Enzymes in the Striatum and in the Substantia Nigra in Mice after MPTP Administration

**Figure 8:**
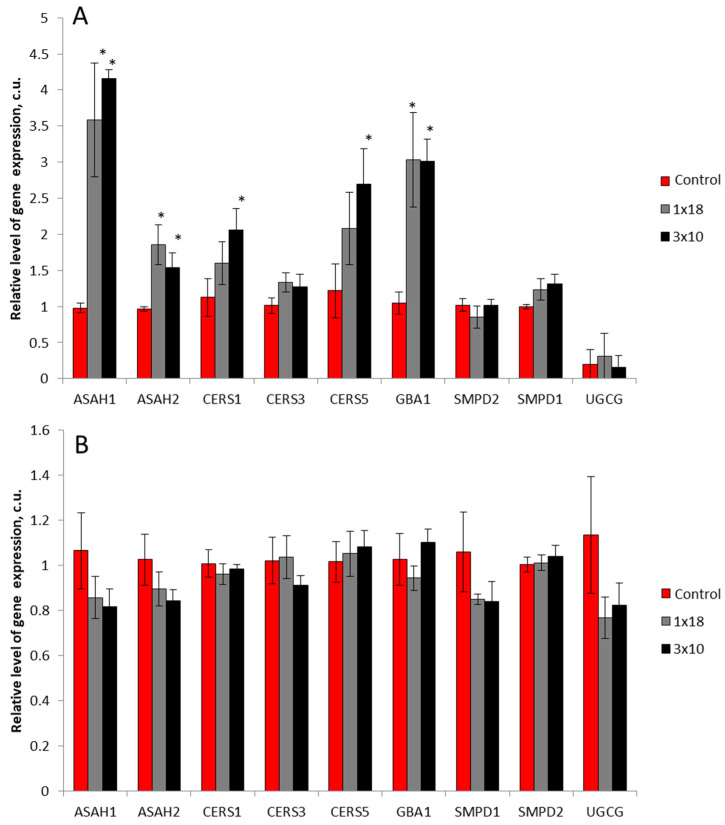
Relative level of gene expression in the substantia nigra (**A**) and in the striatum (**B**) in mice 2 weeks after the administration of 1-methyl-4-phenyl-1,2,3,6-tetrahydropyridine (MPTP): once at a single dose of 18 mg/kg (1 × 18) or three times at a single dose of 10 mg/kg (3 × 10), with a 2-h interval between injections, as well as after saline administration in the controls. ASAH1, acid ceramidase; ASAH2, neutral ceramidase; CERS1, CERS3, CERS5, ceramide synthases 1, 3, and 5, respectively; GBA1, glucocerebrosidase; SMPD1, sphingomyelin phosphodiesterase 1 (acid sphingomyelinase); SMPD2, sphingomyelin phosphodiesterase 2 (neutral sphingomyelinase); UGCG, ceramide glucosyltransferase. * *p* < 0.05, significant differences between the experiment and the controls.

When analyzing the expression of nine genes of sphingolipid metabolism enzymes (ASAH1, acid ceramidase; ASAH2, neutral ceramidase; CERS1, CERS3, CERS5, ceramide synthases 1, 3, 5, respectively; GBA1, glucocerebrosidase; SMPD1, sphingomyelin phosphodiesterase 1 (acid sphingomyelinase); SMPD2, sphingomyelin phosphodiesterase 2 (neutral sphingomyelinase); UGCG, ceramide glucosyltransferase) in the SN in mice 2 weeks after MPTP injection, changes in the expression of five genes were found: CERS1, CERS5, ASAH1, ASAH2, and GBA1 (Figure 6A). In all five cases, gene expression in the mice treated with MPTP was higher than in the controls after saline administration. The expression of the ASAH1, ASAH2, and GBA1 genes changed equally in mice that received MPTP once or three times, while the expression of the CERS1 and CERS5 genes increased in mice only after a triple injection of MPTP. However, the degree of changes in the level of gene expression varied significantly. Thus, the expression of the ASAH1 gene increased by 3.5 times, that of ASAH2 and CERS5 increased by 1.7 times and 2.3 times, respectively, while the respective concentrations of CERS1 and GBA1 increased twofold (Figure 8A).

In contrast to the substantia nigra, no changes in the expression of any of the studied genes were found in the striatum (Figure 8B).

## 4. Discussion

### 4.1. Modeling Parkinson’s Disease

Our recent study [27] was one of the first to focus on changes in sphingolipid content in the nigrostriatal system, striatum and the SN, in mice with MPTP modeling of the advanced (late) clinical stage of PD (four subcutaneous injections of MPTP at a single dose of 10 mg/kg with an interval of 2 h between injections). In that study, we showed the great potential of this approach for assessing changes in sphingolipid metabolism in the pathogenesis of PD. The main advantage of the MPTP model of PD over other genetic and neurotoxic models is the opportunity to reproduce the systemic and stagewise development of PD [28]. This is due to the fact that MPTP is converted in the body into MPP+, a toxin of catecholaminergic neurons, which causes the death of these neurons in the brain, as well as in the peripheral nervous system in animals, which is also typical for patients [29]. Our MPTP models of PD at the preclinical and clinical stages were developed 10 years ago [28] and then used to assess the stage-dependent molecular mechanisms of neurodegeneration and neuroplasticity in the nigrostriatal dopaminergic system [25,30,31], as well as the peripheral manifestations of these processes [32,33].

Given that the strategic task faced by neurologists is to develop early (preclinical) diagnosis and preventive neuroprotective treatment of PD [3], it is of particular interest to gain new fundamental knowledge of the molecular mechanisms of PD pathogenesis at the preclinical stage and during the transition from the preclinical stage to the clinical stage. Proceeding from the fact that the first motor symptoms in patients appear after a decrease in the level of dopamine in the striatum to a threshold of 30–20% [34], in this study, we selected those schemes of MPTP use in which the late preclinical (prodromal) stage and the early clinical stage of PD are reproduced. To reproduce the preclinical stage, the animals were injected once subcutaneously with MPTP at a dose of 18 mg/kg. This led to a pre-threshold decrease in the level of dopamine in the striatum (by 60%) without impairing motor behavior. To reproduce the early clinical stage, MPTP was administered to the animals three times at a single dose of 10 mg/kg with 2 h intervals between injections. This led to a threshold decrease in dopamine levels (by 82%) and motor disorders. Based on our earlier work, the loss of dopaminergic neurons in the SN was approximately 25% [28].

### 4.2. The Content of Sphingolipids in the Striatum and Substantia Nigra in Models of Parkinson’s Disease and Assessment of Their Possible Role in the Pathogenesis

**Figure 9:**
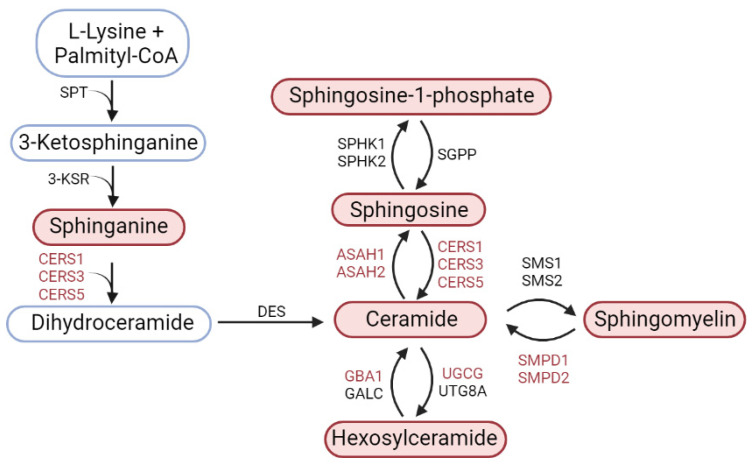
Scheme for the synthesis of sphingolipids. Lipids and enzymes that were examined in this study are marked in red. UGCG, ceramide glucosyltransferase; CERS1, CERS3, CERS5, ceramide synthases 1, 3, and 5, respectively; ASAH1, acid ceramidase; ASAH2, neutral ceramidase; GBA1, glucocerebrosidase; SMPD1, sphingomyelin phosphodiesterase 1 (acid sphingomyelinase); SMPD2, sphingomyelin phosphodiesterase 2 (neutral sphingomyelinase); SPT, serine-palmitoyl transferase; 3-KSR, 3-ketosphinganine reductase; DES, dihydroceramide desaturase; GALC, galactosyceramidase; UTG8A, 1-beta-galactosyltransferase; SPHK1, sphingosine kinase 1; SPHK2, sphingosine kinase 1; SGPP, sphingosine-1-phosphate phosphatase; SMS1, sphingomyelin synthase 1; SMS2, sphingomyelin synthase 2.

In our MPTP models of preclinical and clinical stages of PD, we measured the concentration of sphingolipids, such as ceramides, sphingomyelins, monohexosylceramides, sphinganine, sphingosine, and sphingosine-1-phosphate, in the striatum and SN in mice using mass spectrometry (Figure 9). In neurodegenerative diseases, including PD, these sphingolipids are involved in a wide range of pathological processes: neuroinflammation, neurodegeneration, apoptosis, and autophagy [10,22]. However, under certain conditions, these substances can stimulate reparative processes [10,22].

According to our data, in the striatum of control mice, the total level of ceramides (the basic family of sphingolipids) is approximately twice as high as in the SN. Of the 11 molecular species of ceramides we measured, the concentration of only one of them changed (increased) in PD models, 16-0, and only in the SN in mice in the PD preclinical-stage model (Table S1). Surprisingly, this change was leveled out at the early clinical stage. However, as shown in our previous research, the concentration of ceramides in the SN doubles in the advanced clinical-stage model, as the level of three types of ceramides rises then: d18:1/16:0, d18:1/18:0, and d18:1/24:1 [27]. Our data in this and previous studies are consistent with the general idea that intracellular accumulation of ceramides is one of the triggers and modulators of neurodegeneration [35].

Some of the most functionally significant derivatives of ceramides, which are also among the most widely represented in the nigrostriatal system, are sphingomyelins (Figure 9). The general level of sphingomyelins in the controls in the striatum practically did not differ from the level in the SN. Of particular interest is the fact that the total concentration of sphingomyelins in the striatum and SN exceeded the level in the controls only in the preclinical-stage model (Table 2).

Indeed, in the model of the early clinical stage, this indicator does not differ from that in the controls. The increase in the total concentration of sphingomyelins measured by us in the striatum in the preclinical-stage model is due to an increase in the concentration of sphingomyelin 20-0, and in the SN, this is due to an increase in the concentration of sphingomyelins 16-0, 18-0, and 20-0 (Table 2). Taken together, these data suggest that sphingomyelins are involved in reparative rather than neurodegenerative processes.

The data obtained in this study in the MPTP model of the early clinical stage of PD are in good agreement with the absence of changes in the total concentration of sphingomyelins shown earlier in the MPTP model of the advanced clinical stage of PD [27]. It seems that the MPTP models we used reproduce well the changes in sphingomyelin metabolism in PD patients. Indeed, in patients (men and women), there are no differences in the level of sphingomyelins in the striatum (putamen) and in the SN in comparison with the age-matched controls, although the content of sphingomyelins in the SN was different compared to the controls in women only [36,37]. In contrast to women, in men, the level of sphingomyelins in the SN increases [37]. The accumulation of sphingomyelins in the human brain in various pathologies usually occurs due to a mutation of the sphingomyelinase 1 gene, which is considered a risk factor for the development of PD [38]. This is primarily due to the fact that sphingomyelins stimulate the aggregation of alpha-synuclein, as evidenced, in particular, by the inclusion of sphingomyelins in Lewy bodies [39,40].

The third group of sphingolipids, which is involved in the metabolism of ceramides, is represented by sphinganine, sphingosine, and sphingosine-1-phosphate (Figure 9). In the pathogenesis of PD, sphingosine induces the formation of oligomeric complexes of alpha-synuclein in the neurons of mammals, including humans [41]. In turn, alpha-synuclein suppresses the expression and activity of sphingosine kinase 1, an enzyme that catalyzes the phosphorylation of sphingosine to S1P [42] and modulates signaling through the S1P receptor [10,43]. In various neurotoxic PD models using MPTP, 6-HDA, and rotenone, it was shown that S1P can play a neuroprotective role by reducing the aggregation of alpha-synuclein [43,44,45,46]. However, the data obtained in clinical and experimental studies do not yet allow us to draw definite conclusions about the role of sphingosine and its derivatives in the pathogenesis of PD.

Finally, the fourth group of sphingolipids analyzed in this study includes several molecular types of monohexosylceramides: 18-0, 20-0, 22-0, 24-0, and 24-1. In contrast to most of the analyzed molecular forms of sphingolipids (ceramides, sphinganine, sphingosine, and sphingosine-1-phosphate), the concentration of hexosylceramide 20-0 (Table 2) changed (increases) in the striatum in the model of the preclinical stage of PD and remained elevated in mice in the clinical-stage model compared to the controls. In addition, in the striatum of mice in the model of clinical PD, the concentration of hexosylceramide 24-1 increased. This led to an increase in the total concentration of hexosylceramides in the striatum in the early clinical-stage model (Table 2). When comparing the data obtained in this study (Table 2) and in our previous research [27], it can be assumed that an increase in the level of monohexosylceramides can be expected in patients but only at the advanced clinical stage of PD, as seen in the corresponding MPTP models [27]. As for the role of monohexosylceramides in the pathogenesis of PD, data on this issue are extremely limited in existing published research. Nevertheless, it is believed that monohexosylceramides promote the dissociation of aggregated complexes of alpha-synuclein with the accompanying formation of toxic monomers [47]. Interestingly, aging in mice is accompanied by the accumulation of both glucosylceramide and lactosylceramide in the brain, which is assumed to occur in PD as well [48].

### 4.3. Expression of Genes of Key Sphingolipid Metabolism Enzymes in Models of Parkinson’s Disease

Some clarity in understanding the mechanisms responsible for changes in sphingolipid metabolism can be achieved by assessing changes in enzyme systems that catalyze the metabolic transformations of sphingolipids. Indeed, it has been shown that changes in the expression and/or activity of enzymes of sphingomyelin metabolism can serve as one of the triggers of PD development. Such changes include a mutation in the GBA gene encoding glucocerebrosidase, which cleaves glucosylceramide to glucose and ceramide [49]. A decrease in GBA activity leads to lysosomal dysfunction and accumulation of glucosylceramide in lysosomes, which stabilizes the toxic oligomeric form of alpha-synuclein. At the same time, alpha-synuclein inhibits the activity of GBA, which aggravates the toxic effect of this protein on neurons [47]. The role of other sphingolipid metabolism enzymes is extremely poorly understood.

This study is the first attempt to carry out a comprehensive analysis of changes in the gene expression of nine key enzymes of sphingolipid metabolism, mainly ceramides (Figure 9). The enzymes we chose catalyze the conversion of sphingomyelins (SMPD1, SMPD2) and monohexosylceramides (GBA1) into ceramides, as well as the interconversion of ceramides and sphingosine (CERS1, CERS3, CERS5, ASAH1, and ASAH2). It should be noted that CERS1 is mainly involved in the synthesis of ceramides containing C18 fatty acyl chains. CERS3 is responsible for the synthesis of ceramides containing C18:0 and C24:0 fatty acyl chains. CERS5 is involved predominantly in the formation of ceramides containing C14:0 and C16:0 fatty acid chains. To understand the role played in the metabolism of sphingolipids by those enzymes whose gene expression changes in PD models, the features of these changes in various parts of the nigrostriatal system, the striatum and the SN, may be of great importance.

When modeling the preclinical and early clinical stages of PD by administering MPTP to mice, we did not observe changes in the expression of all the studied genes in the striatum. On the contrary, when modeling both stages of PD, the expression of five genes of the nine selected genes of sphingolipid metabolism enzymes changed (increased) in the SN: ASAH1, ASAH2, CERS1, CERS5, and GBA1. Based on these data and considering the fact that in the similar MPTP models we used previously, neurodegenerative and reparative processes occurred both in the striatum (in the place of projection of dopaminergic axons) and in the SN (in the area of localization of dopaminergic cell bodies) [25,30,31], it can be assumed that the genes analyzed by us mainly belong to dopaminergic neurons rather than glial cells.

According to our data, in the SN of mice in the models of both preclinical and clinical stages of PD, the expression of three genes increases: ASAH1, ASAH2, and GBA1. In this case, the expression of the ASAH1 gene increases 3.5 times as compared to the controls, the expression of the ASAH2 gene increases almost twofold, and the expression of the GBA1 gene increases threefold. In contrast to the ASAH1, ASAH2, and GBA1 genes, the expression of the CERS1 and CERS5 genes increases only in the model of the clinical stage of PD. However, there is no simultaneous increase in the content of ceramides. This may be due to their rapid enzymatic conversion to sphingosine or sphingomyelins (Figure 9). In turn, sphingosine, as a result of phosphorylation, is converted into S1P, which has neuroprotective properties [50]. Our data confirm the advisability of using inhibitors of enzymes involved in the metabolism of glucosylceramides for the treatment of PD [51,52].

## 5. Conclusions

In this study, it was shown for the first time on animal MPTP models of the preclinical and clinical stages of PD that the stepwise development of PD is accompanied by changes in the expression of genes of key sphingolipid metabolism enzymes, significantly only in the SN, the area of localization of the nigrostriatal dopaminergic neurons (cell bodies), and not in the striatum, the area of projection of the axons of dopaminergic neurons. The changes in the expression of genes of sphingolipid metabolism enzymes in the SN are accompanied by changes in the metabolism of sphingolipids, both in the SN and in the striatum. The greatest changes in the content of sphingolipids were found in mice in the model of the preclinical stage of PD, when reparative processes dominate over neurodegenerative processes. Changes of this kind, found in patients with other brain diseases, are considered factors that trigger or modulate neurodegenerative and reparative processes. Our pioneering study of changes in the expression of genes of sphingolipid metabolism enzymes and concomitant changes in the content of sphingolipids in the nigrostriatal system in mice using models of the preclinical and clinical stages of PD opens up broad prospects for the development of innovative technologies for early diagnosis and preventive treatment of PD based on the obtained fundamental knowledge.

## Figures and Tables

**Table 1:** List of primers used for real-time PCR.

Gene	Forward Primer	Reverse Primer
ASAH1	GCAGAAAATCAACGTATCCTCC	ATTCCCCTCATTTCCTCTCC
ASAH2	ACACCCTGTCTGCATACATC	TCAACCACTTCTCCCACTC
GBA1	TATCCAACTTCAGCCTCCC	CCCCTTCCCATTCACTCTTC
SMPD1	ATCAGTCAACCACAACGAAG	GTCAAACAGAGCCAGAACC
SMPD2	CATCCACCACACATCCAAG	TACAGCCATCATCAGAGCC
CERS1	TGCCACTCCATCTATGCCAC	GGAACCAGAACCAGCAGAAAC
CERS3	CCCTTCTTCTCCTACATCTTCC	TCCTCTTCCTCCTCCTCTTC
CERS5	ACAACCCACAAAAACAACCTC	CACACACAAAAACCACACAAAC
UGCG	TCCTTTCCCCTCCTTTTCC	CATCCACCAAACTCCACTAAAC

**Table 2:** Summary table of changes in sphingolipid content and changes in the gene expression of sphingolipid metabolism enzymes in mice in the striatum and in the substantia nigra 2 weeks after subcutaneous administration of MPTP: once at a single dose of 18 mg/kg and three times at a single dose of 10 mg/kg with a 2-h interval between injections compared to the controls (saline).

**Sphingomyelins**														
**Brain Structure**	**Animal Group**	**14-0**	**16-1**	**16-0**	**18-1**	**18-0**	**20-1**	**20-0**	**22-1**	**22-0**	**24-1**	**24-0**	**26-1**	**Total (** **Cumulative) Concentration**
Striatum	1 × 18							↑						↑
	3 × 10													
SN	1 × 18			↑		↑		↑						↑
	3 × 10													
**Ceramides**														
**Brain Structure**	**Animal Group**	**16-1**	**16-0**	**18-1**	**18-0**	**20-1**	**20-0**	**22-1**	**24-1**	**24-0**	**26-1**	**26-0**	**Total (Cumulative) Concentration**	
Striatum	1 × 18													
	3 × 10													
SN	1 × 18		↑											
	3 × 10													
**Hexosylceramides**														
**Brain Structure**	**Animal Group**	**18-0**		**20-0**		**22-0**			**24-0**		**24-1**		**Total (Cumulative) Concentration**	
Striatum	1 × 18			↑										
	3 × 10			↑							↑		↑	
SN	1 × 18													
	3 × 10													
**Derivatives of Ceramides**														
**Brain Structure**			**Animal Group**			**Sphingosine**			**Sphinganine**			**Sphingosine-1-phosphate**		
Striatum			1 × 18											
			3 × 10											
SN			1 × 18									↑		
			3 × 10									↑		
**Changes in Gene Expression**														
**Brain Structure**	**Animal Group**	**ASAH1**		**ASAH2**	**CERS1**		**CERS3**	**CERS5**		**GBA1**	**SMPD1**		**SMPD2**	**UGCG**
Striatum	1 × 18													
	3 × 10													
SN	1 × 18	↑		↑						↑				
	3 × 10	↑		↑	↑			↑		↑				

↑ = increase compared to the controls.

## Data Availability

The data presented in this study are available on request from the corresponding author. The data are not publicly available due to legal issues.

## Supplementary Materials

The following are available online at https://www.mdpi.com/article/10.3390/biom12010093/s1, Table S1. Concentrations (ng/10 mg tissue) of different molecular forms of sphingomyelins in the striatum and in the substantia nigra in mice two weeks after: the administration of 1-methyl-4-phenyl-1,2,3,6-tetrahydropyridine (MPTP) once at a single dose of 18 mg/kg (1 × 18), the administration of MPTP three times at a single dose of 10 mg/kg with a two-hour interval between injections (3 × 10), as well as after the of saline (control). The table shows mean values and standard error of the mean. * *p* < 0.05, significant differences compared to the control; # *p* < 0.05, significant differences between the groups 1 × 18 and 3 × 10. Table S2. Concentrations (ng/10 mg tissue) of different molecular forms of ceramides in the striatum and in the substantia nigra in mice two weeks after: the administration of 1-methyl-4-phenyl-1,2,3,6-tetrahydropyridine (MPTP) once at a single dose of 18 mg/kg (1 × 18), the administration of MPTP three times at a single dose of 10 mg/kg with a two-hour interval between injections (3 × 10), as well as after the administration of saline (control). The table shows mean values and standard error of the mean. * *p* < 0.05, significant differences compared to the control; # *p* < 0.05, significant differences between the groups 1 × 18 and 3 × 10. Table S3. Concentrations (ng/10 mg tissue) of different molecular types of hexosylceramides in the striatum and in the substantia nigra in mice two weeks after: the administration of 1-methyl-4-phenyl-1,2,3,6-tetrahydropyridine (MPTP) once at a single dose of 18 mg/kg (1 × 18), the administration of MPTP three times at a single dose of 10 mg/kg with a two-hour interval between injections (3 × 10), as well as after the administration of saline (control). The table shows mean values and standard error of the mean. * *p* < 0.05, significant differences compared to the control.

## Abbreviations

PD	Parkinson’s disease
SN	substantia nigra
MPTP	1-methyl-4-phenyl-1,2,3,6-tetrahydropyridine
UGCG	ceramide glucosyltransferase
CERS1	ceramide synthase 1
CERS3	ceramide synthase 3
CERS5	ceramide synthase 5
ASAH1	acid ceramidase
ASAH2	neutral ceramidase
GBA1	glucocerebrosidase
SMPD1	sphingomyelin phosphodiesterase 1 (acid sphingomyelinase)
SMPD2	sphingomyelin phosphodiesterase 2 (neutral sphingomyelinase)
