# Supplementary material for: The Sphingolipid Asset Is Altered in the Nigrostriatal System of Mice Models of Parkinson’s Disease
Source: Biomolecules. 2022 Jan 6;12(1):93. doi: 10.3390/biom12010093 (PMC8773707; doi:10.3390/biom12010093)
Supplement: Supplementary file 1 [file biomolecules-12-00093-s001.zip › Tables S1 S2 S3 Blokhin 25 12 21 Biomolecules.pdf]

Table S1. Concentrations (ng/10 mg tissue) of different molecular forms of sphingomyelins in the striatum and in the substantia nigra in mice two weeks after: the administration of 1-methyl-4-phenyl-1,2,3,6-tetrahydropyridine (MPTP) once at a single dose of 18 mg/kg (1x18), the administration of MPTP three times at a single dose of 10 mg/kg with a two-hour interval between injections (3x10), as well as after the of saline (control). The table shows mean values and standard error of the mean. *p <0.05, significant differences compared to the control; # p <0.05, significant differences between the groups 1x18 and 3x10.

	Sphingomyelins / Groups	14-0	16-1	16-0	18-1	18-0	20-1	20-0	22-1	22-0	24-1	24-0	26-1	Total concentration
**Striatum**	**Control**	37.51 (2.63)	33.52 (1.95)	671.36 (22.89)	1917.46 (109.5)	12595.22 (319.91)	67.85 (3.02)	2625.85 (69.53)	207.96 (9.52)	13.57 (0.02)	1820.82 (78.49)	400.48 (15.15)	24.59 (1.04)	***20389.06 (469.25)***
	**1x18**	32.41 (0.86)	32.65 (1.83)	684.28 (39.77)	2082.71 (173.79)	13741.3 (613.66)	60.79 (3.16)	**3794.40* (137.36)**	228.85 (14.20)	13.63 (0.04)	1648.24 (131.18)	457.41 (28.18)	24.58 (0.64)	***22801.25* (895.48)***
	**3x10**	31.93 (1.05)	31.54 (1.29)	678.83 (35.44)	1894.20 (128.80)	**11922.8# (264.11)**	59.41 (3.06)	**2780.12#** (79.81)	231.86 (11.34)	13.63 (0.03)	**2280.31# (183.19)**	547.41 (53.27)	19.95 (4.57)	***20491.94# (618.58)***
**SN**	**Control**	35.31 (0.95)	27.85 (0.75)	493.35 (41.63)	1380.62 (71.99)	11687.92 (479.81)	75.70 (2.60)	2230.76 (106.93)	399.13 (18.51)	14.05 (0.05)	5915.17 (366.41)	1441.18 (102.52)	57.06 (4.21)	***23758.11 (1149.1)***
	**1x18**	36.09 (1.25)	28.63 (1.21)	**651.91*** (26.10)	1587.92 (73.64)	**13203.9* (335.08)**	87.73 (7.38)	**3346.73*** (104.37)	468.65 (30.85)	14.23 (0.08)	6299.35 (337.01)	1497.04 (82.37)	50.31 (2.79)	***27241.58* (713.77)***
	**3x10**	35.91 (0.98)	28.83 (0.50)	591.76 (17.77)	1575.80 (62.03)	12756.55 (315.85)	76.78 (2.67)	**2529.77#** (142.19)	413.89 (12.68)	14.09 (0.03)	5989.51 (260.24)	1446.37 (63.25)	61.04 (3.43)	***25520.31 (712.38)***

Table S2. Concentrations (ng/10 mg tissue) of different molecular forms of ceramides in the striatum and in the substantia nigra in mice two weeks after: the administration of 1-methyl-4-phenyl-1,2,3,6-tetrahydropyridine (MPTP) once at a single dose of 18 mg/kg (1x18), the administration of MPTP three times at a single dose of 10 mg/kg with a two-hour interval between injections (3x10), as well as after the administration of saline (control). The table shows mean values and standard error of the mean. *p <0.05, significant differences compared to the control; # p <0.05, significant differences between the groups 1x18 and 3x10.

Groups	Ceramides	14-0	16-0	16-1	18-0	18-1	20-0	22-0	22-1	24-0	24-1	26-0	26-1	Total concentration
**Striatum**	**Control**	8.73 (1.65)	45.55 (5.36)	1.21 (0.07)	1078.46 (94.29)	3.78 (0.22)	40.00 (2.66)	24.11 (1.96)	8.67 (0.90)	19.19 (2.16)	103.46 (14.04)	6.30 (0.28)	1.42 (0.06)	***1340.87 (122.65)***
	**1x18**	11.47 (2.22)	48.50 (6.64)	1.32 (0.08)	1185.41 (186.01)	3.69 (0.35)	38.35 (4.86)	26.28 (2.03)	9.77 (1.57)	17.63 (1.57)	106.43 (17.75)	7.04 (0.32)	1.38 (0.04)	***1457.27 (214.23)***
	**3x10**	9.60 (1.08)	50.02 (6.29)	1.21 (0.07)	1024.16 (98.83)	3.73 (0.31)	38.95 (2.83)	21.72 (1.50)	9.30 (0.99)	21.19 (1.48)	122.13 (13.04)	7.41 (0.54)	1.56 (0.08)	***1310.99 (120.89)***
**SN**	**Control**	6.56 (0.95)	15.90 (2.65)	1.22 (0.13)	479.57 (49.15)	1.86 (0.13)	22.17 (0.84)	21.72 (1.50)	7.98 (0.38)	22.86 (2.25)	120.39 (16.28)	7.54 (0.52)	1.76 (0.07)	***709.52 (73.03)***
	**1x18**	5.53 (1.44)	**23.2* (1.19)**	1.21 (0.15)	572.86 (47.44)	1.95 (0.12)	22.32 (0.61)	25.65 (2.13)	8.71 (0.40)	25.77 (4.85)	133.68 (10.96)	6.90 (0.82)	1.67 (0.13)	***827.80 (59.14)***
	**3x10**	4.86 (0.77)	19.21 (1.35)	1.18 (0.15)	606.13 (39.57)	2.18 (0.08)	24.28 (1.08)	23.11 (0.85)	8.64 (0.19)	19.46 (0.61)	128.24 (7.30)	6.35 (0.41)	1,56 (0.06)	***845.20 (41.87)***

Table S3. Concentrations (ng/10 mg tissue) of different molecular types of hexosylceramides in the striatum and in the substantia nigra in mice two weeks after: the administration of 1-methyl-4-phenyl-1,2,3,6-tetrahydropyridine (MPTP) once at a single dose of 18 mg/kg (1x18), the administration of MPTP three times at a single dose of 10 mg/kg with a two-hour interval between injections (3x10), as well as after the administration of saline (control). The table shows mean values and standard error of the mean. *p <0.05, significant differences compared to the control.

Groups	Hexosylceramides	HexCer 18-0	HexCer 20-0	HexCer 22-0	HexCer 24-0	HexCer 24-1	Total concentration
Striatum	Control	51.21 (2.56)	7.33 (0.28)	48.10 (1.86)	72.90 (3.54)	180.15 (8.27)	*359.68 (14.63)*
	1x18	58.93 (4.72)	9.57* (0.70)	50.56 (2.67)	85.54 (7.77)	212.91 (14.12)	*385.24 (49.22)*
	3x10	61.23 (5.37)	8.96* (0.27)	55.62 (2.67)	90.23 (5.32)	220.74* (10.78)	*436.78* (23.21)*
SN	Control	100.79 (9.59)	15.87 (0.63)	91.86 (3.95)	166.69 (9.60)	434.08 (17.29)	*809.30 (40.18)*
	1x18	117.42 (6.90)	16.26 (0.78)	89.33 (3.37)	182.71 (7.29)	491.78 (20.43)	*897.51 (34.77)*
	3x10	116.14 (6.40)	16.00 (0.78)	96.70 (4.10)	179.81 (9.07)	470.41 (19.37)	*879.05 (36.43)*
